# Salivary Gland FNA Diagnostics in a Real-Life Setting: One-Year-Experiences of the Implementation of the Milan System in a Tertiary Care Center

**DOI:** 10.3390/cancers11101589

**Published:** 2019-10-18

**Authors:** Erkka Tommola, Satu Tommola, Sinikka Porre, Ivana Kholová

**Affiliations:** 1 Department of Pathology, Fimlab Laboratories, Tampere University Hospital, 335 20 Tampere, Finland; erkka.tommola@tuni.fi (E.T.); satu.tommola@fimlab.fi (S.T.); sinikka.porre@fimlab.fi (S.P.); 2 Faculty of Medicine and Health Technology, Tampere University, 33520 Tampere, Finland

**Keywords:** salivary glands, FNA, The Milan System for Reporting Salivary Gland Cytopathology

## Abstract

The Milan System for Reporting Salivary Gland Cytopathology (MSRSGC) was introduced in 2018 following other organ specific cytopathological reporting systems and it aimed at bringing a practical, evidence-based, user-friendly classification system with characterization and management algorithms. At the Department of Pathology, Fimlab Laboratories, Tampere, Finland all salivary fine needle aspirations (FNAs) have been given cytopathological diagnoses according to the MSRSGC since January 2018. Analyses of a one-year-period (January 2018–December 2018) consisted of 183 salivary FNA samples from 138 patients with correlation to histopathology in 90 cases with surgical follow-up. The MSRSGC performance in patient based analysis was as follows: accuracy was 90.9%, sensitivity was 61.5%, specificity was 100%, positive predictive value was 100%, and negative predictive value was 89.4%, respectively. Risks of malignancy (ROMs) in MSRSGC categories were: 0.0% (0/15) in non-diagnostic category, 100.0% (1/1) in non-neoplastic category biased by only one falsely-negative lymphoma case, 14.3% (1/7) in atypia of undetermined significance category, 0.0% (0/28) in benign neoplasm category, 27.3% (3/11) in neoplasm of uncertain malignant potential category, and 100% for both suspicious for malignancy (4/4) and malignancy (4/4) categories, respectively. The MSRSGC has been proven as a reliable classification system in salivary gland FNA routine diagnostics in a tertiary care center.

## 1. Introduction

The Milan System for Reporting Salivary Gland Cytopathology (MSRSGC) was introduced in 2018 [1] following a successful wave of organ specific reporting systems [2]. The initial internet survey among cytopathologists demonstrated strong support for the new classification system [3]. The goal of the MSRSGC was to develop a practical, evidence-based, user-friendly, and internationally accepted classification system. The system includes characterization and management algorithms for each category [1,4]. Recently, the clinicians endorsed the routine use of the MSRSGC [5].

Salivary gland tumors are one of the most heterogeneous groups of neoplasms with cytopathological features overlapping among the entities. Due to those facts, salivary gland cytopathology is one of the most challenging areas of cytology [6]. Nevertheless, the effectiveness of salivary gland fine needle aspirations (FNA) was shown, as follows: sensitivity 86%, specificity 92%, and diagnostic accuracy 90% [7]. A recent meta-analysis of 16 456 cases from 92 studies confirmed that FNA as a useful method in the salivary gland diagnostics. Furthermore, the retrospective application of the MSRSGC showed an increase in the FNA reliability and reproducibility [8]. 

The aim of the present study was to analyze salivary gland FNA diagnostics after the implementation of the MSRSGC in the practice of a tertiary care center serving a university hospital, regional hospitals, community health care centers, and private practices.

## 2. Results

During a one-year-period, 183 salivary gland FNA samples were diagnosed from 138 patients, consisting of 64 (46.4%) males and 74 (53.6%) females. Table 1 shows the clinical characteristics of the cases in each diagnostic category (Table 1). The average age was higher in those who did not have surgical follow-up than those who did. Additionally, the average age was higher in those who had received malignant cytological diagnoses. Lesions with benign cytological diagnoses were bigger in cases with surgical follow-up (average of 2.3 cm vs. 2.0 cm), but lesions with malignant cytological diagnoses were smaller in cases with surgical follow-up (1.5 cm vs. 2.8 cm). In the present study, 153 (83.6%) samples were from the parotid gland, 25 (13.7%) were from the submandibular gland, and five were from other areas: four from parotid lymph nodes and one from the parotid gland area.

A repeat FNA was taken twice in 29 (21.0%) patients, FNA was taken three times in six (4.3%) patients, and FNA was taken four times in two (1.4%) patients. Among all 37 patients with repeat FNA, the 1^st^ FNA was 23× Non-Diagnostic, 8× Atypia of Undetermined Significance (AUS), 4× Neoplasm of Uncertain Malignant Potential (SUMP), in one case Benign Neoplasm and in one case Suspicious for Malignancy. Out of these 37 patients with repeated FNAs, the cytopathological diagnosis changed in 15 (40.5%) cases: in nine cases from Non-Diagnostic category 1× to Non-Neoplastic, 2× to Atypia of Undetermined Significance (AUS), 3× to Benign Neoplasm, 2× to Neoplasm of Uncertain Malignant Potential (SUMP), and 1× to Malignant Neoplasm. In six cases, the cytopathological diagnosis changed from Atypia of Undetermined Significance (AUS) category 3× to Non-Diagnostic and 3× to Neoplasm of Uncertain Malignant Potential (SUMP).

In 90 (49%) FNA cases corresponding to 70 patients, histological follow-up was available, and Table 2 shows all histological findings according to MSRSGC categories (Table 2). In histologically confirmed cases, 50 cases belonged to benign MSRSGC categories (Non-Neoplastic, Atypia of Undetermined Significance, Benign Neoplasm and Neoplasm of Uncertain Malignant Potential). The cytological diagnosis was true-negative in 45 of 90 cases and false-negative in five of 90 cases. In Neoplasm of Uncertain Malignant Potential (SUMP) category, three of 12 (25.0%) cases turned out to be malignant. All three patients were given cytological diagnoses cellular pleomorphic adenoma, but turned out to be carcinoma ex pleomorphic adenoma in histopathology. One out of two non-SUMP false-negative cases was diagnosed as reactive changes on cytology and was categorized into Non-Neoplastic category, but turned out to be extranodal marginal zone B-cell lymphoma of MALT type. The other non-SUMP false-negative case was given a cytological diagnosis as AUS, but it was reported as adenoid cystic carcinoma on histology.

There were nine out of 90 (10%) cases that belonged to the category defined as malignant (Suspicious for Malignancy and Malignant Neoplasm) and all of them turned out to be true-positive cytological diagnoses and none of them were false-positively diagnosed.

Thirty-one (34.4%) of FNA sample cases belonged to the Non-Diagnostic category and 11 of 31 (35.4%) cases turned out to be benign neoplasm and two (6.5%) cases turned out to be malignant neoplasms (extranodal marginal zone B-cell lymphoma of MALT type and carcinoma ex pleomorphic adenoma).

Evaluation of MSRSGC was conducted both patient and sample based for the cases that had histopathological follow-up, as shown in Table 3 (Table 3). When reviewing the results patient based, sensitivity and specificity were 61.5% and 100%, respectively. The diagnostic accuracy of FNA for differentiating between benign and malignant disease was 90.9%. In the sample based evaluation, a slightly higher sensitivity (64.3%) and accuracy (91.5%) were observed. With both evaluation perspectives, the positive predictive value was 100%. The negative predictive values were in the patient based and in the sample based evaluation 89.4% and 90.0%, respectively. The results were also separately calculated for the parotid gland and the submandibular gland. In the comparison between lesions in the parotid gland and the submandibular gland, respectively, the accuracy was 94.0% vs. 71.4%; sensitivity 66.7% vs. 50.0%; and, specificity 100% in both.

Among all FNAs with histological follow-up, 22 out of 90 cases (24.4%) were non-neoplastic lesions, whereas 68 lesions (75.6%) were neoplastic and 16 lesions (17.8%) were malignant. The risk of neoplasm, the risk of malignancy, and the overall risk of malignancy were calculated for each MSRSGC category (Table 3) and compared with other MSRSGC studies [8,9,10,11,12,13,14,15,16,17,18,19,20,21,22,23,24,25,26] and MSRSGC estimated ROMs (Table 4).

## 3. Discussion

The one-year-experience with MSRSGC revealed the diagnostic accuracy of FNA for differentiating between benign and malignant disease to be 90.9%. In a detailed analysis, patient vs. sample based analyses comparison showed slightly higher values in samples based analysis due to the increased amount of true positive and true negative cases in the sample based evaluation. Sensitivity was: 61.5% vs. 64.3%, specificity: 100% vs. 100%, PPV: 100% vs. 100%, and NPV: 89.4% vs. 90.0%, respectively. The lesions with well-established cytopathological features, such as pleomorphic adenoma and Warthin’s tumor, which are the most common salivary gland tumors, were also well represented in our cohort (16 cases of pleomorphic adenoma and 18 cases of Warthin´s tumor) and attributed to high accuracy [6]. In the 19 retrospective studies and meta-analyses that are summarized in Table 4 (Table 4), the overall accuracy was 93.2%, the sensitivity 82.9%, the specificity 95.1%, the PPV 92.0%, and the NPV 92.1%.

As previously noticed, the diagnostic accuracy varies among salivary gland locations [6]. Despite the fact that the parotid glands are the main targets of both benign and malignant neoplasms, 10–15% of all salivary gland tumors affect submandibular glands with 50% of these being malignant. In the presented institutional MSRSGC analysis, accuracy was 94.0% for parotid glands vs. 71.4% for submandibular glands, sensitivity: 66.7% vs. 50.0%, specificity: 100% vs. 100%, PPV: 100% vs. 100%, and NPV: 93.2% vs. 60.0%, respectively. Nevertheless, recently, Maleki et al. showed submandibular gland ROMs of different MSRSGC categories being similar to those reported for parotid gland cytological specimens [15]. In a Finnish study, six out of 18 malignancies in the submandibular glands were false-negative in FNA (Pap classes 0–2) [27].

If MSRSGC categories are analyzed, all benign categories (one clinical exception in NN category) were below MSRSGC estimated ROMs and all malignant categories were above MSRSGC estimated ROMs (Table 4). Interestingly, in contrast to other cytopathological terminologies, MSRSGC also contains a SUMP category specific to salivary glands cytopathology in addition to atypical category (AUS) due to overlapping features among benign and malignant tumors. In our study, the only malignancy that was diagnosed as an AUS case was adenoid cystic carcinoma. In a multi-institutional study, original benign cytological diagnosis was given in 13 out of 46 adenoid cystic carcinoma cases (28.3%) and in MSRSGC reclassification, 23 cases (50%) were diagnosed as SUMP [28]. 

In comprehensive bi-institutional study by Chowsilpa et al., 65 SUMP cases were retrospectively analyzed [29]. The SUMP category RON was 95.4% in comparison to our study 100% RON. In both studies, pleomorphic adenoma was the most common SUMP benign histopathological correlation. Overall, ROM in SUMP category was 33.8% in Chowsilpa et al. [29] study in comparison to 27.3% in our study. In Chowsilpa et al. study, 60% of SUMP cases revealed unspecific features, 20% were basaloid tumors, and 20% were oncocytic tumors with a lowest ROM of 7.7% in comparison to overall 33.8% [29]. Our study consisted only of two cases of oncocytic SUMP tumors being too small sample amount for conclusions. 

False-negative cases were further analyzed to scope diagnostic pitfalls. In our one-year-material, there were five false-negative cases. Extranodal marginal zone B-cell lymphoma of MALT type was falsely diagnosed as reactive lesion in Non-neoplastic MSRSGC category. In a robust review of 6249 cases that was led by the College of American Pathologists, the lymphoma cases featured the highest false-negative rate of 57% [30]. Adenoid cystic carcinoma diagnosed as AUS was discussed in previous paragraph. Three cases of carcinoma ex pleomorphic adenoma were false-negatively diagnosed in the SUMP category. Interestingly, SUMP contained also four other cases of pleomorphic adenoma. Proudly, no cases of false-positive malignancies were reported in the one-year-period.

Unfortunately, we faced high percentage of non-diagnostic cases in our routine practice. In our setting, high percentage of FNAs is taken by a radiologist in training at the beginning of their learning curve shown also by a high percentage of thyroid non-diagnostic FNAs [31]. In histologically confirmed non-diagnostic cases, there were six cystic non-neoplastic lesions and four cystic benign tumors, with both lesions naturally increasing the non-diagnostic rates in cytology [32,33]. In samples-based analysis, two malignancies were originally diagnosed as non-diagnostic, but the diagnostic category increased in repeated FNA. In the literature, ROSE (rapid on site evaluation) also decreased the non-diagnostic rates in salivary gland FNA [34]. According to the Survey on Salivary Gland Cytopathology [3], ROSE is performed by 59% of participants. Retrospective MSRSGC studies and analyses [8,9,10,11,12,13,14,15,16,17,18,19,20,21,22,23,24,25,26] (Table 4) did not scope on the role of ROSE and MSRSGC diagnostic accuracy.

Importantly, an increasing amount of salivary gland tumors harbor genetic mutations or rearrangements also detectable in cytological specimens [35,36,37,38] and the widespread use of ancillary techniques can also increase the diagnostic accuracy and reduce SUMP category amount of cases.

## 4. Materials and Methods 

The Department of Pathology, Fimlab Laboratories, Tampere, Finland began the use of the MSRSGC on January 1st, 2018. In an electronic pathology database all salivary gland cytological diagnoses during a one-year-period (January 1st, 2018–December 31st, 2018) were searched for. The follow-up histopathological reports were included for cases wherever they were available until May 22nd, 2019.

The ultrasound-guided fine needle aspirations (FNA) were performed by radiologists with 22G needles. The specimens were alcohol-fixed, cytospin smears were stained with Papanicolaou stain. Cell blocks were routinely made [39]. All of the FNAs were originally diagnosed according to the MSRSGC.

The MSRSGC was critically evaluated by comparing the preoperative FNA diagnoses with the follow-up histopathological diagnoses. The histological follow-up was used as the gold standard to calculate the risk of neoplasm (RON), the risk of malignancy (ROM), and the overall risk of malignancy (OROM) for each diagnostic category in the MSRSGC. The material was analyzed both patient and sample based. In the patient based analysis the most specific and severe cytopathological diagnosis was used. 

RON in each diagnostic category was calculated as a ratio between neoplastic cases and all cases with histological follow-up. Similarly, ROM was calculated between malignant cases and all cases with histological follow-up. OROM was calculated between malignant cases and all cases with or without histological follow-up.

Suspicious for Malignancy and Malignant Neoplasm categories were considered as true positive findings, while Non-Neoplastic, Atypia of Undetermined Significance, and Neoplasm (both Benign and Uncertain Malignant Potential) categories were considered as true negative findings. The Non-Diagnostic category was excluded when the sensitivity, specificity, positive predictive value (PPV), negative predictive value (NPV), and overall accuracy of FNA for differentiating between benign and malignant disease were determined.

The Ethical Committee of Pirkanmaa Hospital District approved the study (R17174) and informed consent of each individual was not requested. It was conducted according to the Declaration of Helsinki. 

## 5. Conclusions

In conclusions, the presented institutional study provides validation of MSRSGC in salivary gland FNA cytopathological diagnostics in university tertiary care center practice. In our institutional experience, the diagnostic accuracy was 90.9% and ROMs were in agreement with retrospective studies and meta-analyses. 

## Figures and Tables

**Table 1:** Milan System for Reporting Salivary Gland Cytopathology (MSRSGC): Clinical Characteristics, Patient Based.

Cohort	Surgical Follow-Up	Non-Diagnostic	Non-Neoplastic	AUS	Benign Neoplasm	SUMP	Suspicious for Malignancy	Malignant Neoplasm	Total
No. of FNA Cases (%)	Without surgical follow-up	29 (66%)	5 (83%)	5 (42%)	21 (43%)	5 (31%)	1 (20%)	2 (33%)	68 (49%)
	With surgical follow-up	15 (34%)	1 (17%)	7 (58%)	28 (57%)	11 (69%)	4 (80%)	4 (67%)	70 (51%)
Patient Age Average (Range)	Without surgical follow-up	67.4 (26–91)	44.8 (23–69)	62.8 (42–93)	71.3 (45–93)	87.8 (82–90)	66 (66)	76.5 (61–92)	68.4 (23–93)
	With surgical follow-up	58.3 (20–78)	43 (43)	51.4 (25–69)	49.8 (16–71)	65.4 (24–88)	62.5 (48–79)	66.8 (59–73)	55.6 (16–88)
Lesion Size Average (Range)	Without surgical follow-up	1.5 (0.5–5.6)	1.3 (0.9–2.3)	1.5 (0.7–2.2)	2.3 (1.0–3.5)	2.1 (1.0–3.2)	2.5 (2.5)	3 (2.0–4.0)	1.9 (0.5–5.6)
	With surgical follow-up	2.2 (1.0–4.3)	1.4 (1.4)	2.4 (1.8–3.5)	2.4 (0.9–3.2)	2.1 (1.4–5.2)	1.7 (1.0–3.0)	1.2 (1.0–1.7)	2.1 (0.9–5.2)

Abbreviations: AUS, Atypia of Undetermined Significance; SUMP, Neoplasm of Uncertain Malignant Potential.

**Table 2:** Comparison of MSRSGC Diagnoses with Histopathology, Patient Based.

MSRSGC Diagnosis	Histopathology Diagnosis		
	Non-Neoplastic	Beningn Neoplasm	Malignant Neoplasm
Non-Diagnostic	Cyst (*n* = 6)	Warthin’s tumor (*n* = 3)	
	Inflammation (*n* = 4)	Oncocytic cystadenoma (*n* = 1)	
		Lipoma (*n* = 1)	
Non-Neoplastic			Extranodal marginal zone B-cell lymphoma of MALT type (*n* = 1)
AUS	Cyst (*n* = 2)	Warthin’s tumor (*n* = 2)	Adenoid cystic carcinoma (*n* = 1)
	Ractive changes (*n* = 1)	Pleomorphic adenoma (*n* = 1)	
Benign Neoplasm		Warthin’s tumor (*n* = 15)	
		Pleomorphic adenoma (*n* = 12)	
		Eccrine spiradenoma (*n* = 1)	
		Oncocytoma (*n* = 1)	
SUMP		Pleomorphic adenoma (*n* = 4)	Carcinoma ex pleomorphic adenoma (*n* = 3)
		Basal cell adenoma benign (*n* = 1)	
		Oncoytic cystadenoma (*n* = 1)	
		Oncocytoma (*n* = 1)	
Suspicious for Malignancy			Leiomyosarcoma, metastatic site (*n* = 1)
			Myoepithelial carcinoma (*n* = 1)
			Salivary duct carcinoma (*n* = 1)
			Squamous cell carcinoma, metastatic site (*n* = 1)
Malignant Neoplasm			Carcinoma ex pleomorphic adenoma (*n* = 1)
			High grade neuroendocrine carcinoma (*n* = 1)
			Malignant melanoma, metastatic site (*n* = 1)
			Squamous cell carcinoma, metastatic site (*n* = 1)

Abbreviations: AUS, Atypia of Undetermined Significance; SUMP, Neoplasm of Uncertain Malignant Potential.

**Table 3:** Diagnoses According to MSRSGC, Patient and Sample Based.

Based	FNA Category	No./Total No. (%)							
		Cases with Surgical Follow-Up		Risk of Neoplasm		Risk of Malignancy		Overall Risk of Malignancy	
Patient Based	Non-Diagnostic	15/44	(34.1)	5/15	(33.3)	0/15	(0)	0/44	(0)
	Non-Neoplastic	1/6	(16.7)	1/1	(100)	1/1	(100)	1/6	(16.7)
	AUS	7/12	(58.3)	4/7	(57.1)	1/7	(14.3)	1/12	(8.3)
	Benign Neoplasm	28/49	(57.1)	28/28	(100)	0/28	(0)	0/49	(0)
	SUMP	11/16	(68.8)	11/11	(100)	3/11	(27.3)	3/16	(18.8)
	Suspicious for Malignancy	4/5	(80.0)	4/4	(100)	4/4	(100)	4/5	(80.0)
	Malignant Neoplasm	4/6	(83.3)	4/4	(100)	4/4	(100)	4/6	(66.7)
	Total	70/138	(50.7)	57/70	(81.4)	13/70	(18.6)	13/138	(9.4)
Sample Based	Non-Diagnostic	31/74	(41.9)	13/31	(41.9)	2/31	(6.5)	2/74	(2.7)
	Non-Neoplastic	1/6	(16.7)	1/1	(100)	1/1	(100)	1/6	(16.7)
	AUS	9/20	(45.0)	5/9	(55.6)	1/9	(11.1)	1/20	(5.0)
	Benign Neoplasm	28/50	(56.0)	28/28	(100)	0/28	(0)	0/50	(0)
	SUMP	12/21	(57.1)	12/12	(100)	3/12	(25.0)	3/21	(14.3)
	Suspicious for Malignancy	5/6	(83.3)	5/5	(100)	5/5	(100)	5/6	(83.3)
	Malignant Neoplasm	4/6	(66.7)	4/4	(100)	4/4	(100)	4/6	(66.7)
	Total	90/183	(49.2)	68/90	(75.6)	16/90	(17.8)	16/183	(8.7)

Abbreviations: AUS, Atypia of Undetermined Significance; SUMP, Neoplasm of Uncertain Malignant Potential.

**Table 4:** Comparison with Other Studies and MSRSGC Guidelines.

Study			No. of FNABs	No. of Follow-Ups (%)		Yrs	Risk of Malignant (%)							Accuracy	Sensitivity	Specificity	PPV	NPV
							ND	NN	AUS	BN	SUMP	SM	MN	(%)	(%)	(%)	(%)	(%)
MSRSGC estimated ROMs (range)							25	10	20	<5	35	60	>90					
							(0–67)	(0–20)	(10–35)	(0–13)	(0–100)	(0–100)	(57–100)					
Choy 2019 [9]			376	376		14	15	27	29	3	19	88	100	N.D.	N.D.	N.D.	N.D.	N.D.
Farahani 2019 (92 studies) [8]			16456	16456			17	8	34	4	42	58	91	N.D.	96.9	95.3	80.5	97.9
Hang 2018 (12 studies) [10]			1560	694	(44.5)	12	17	10	38	3	41	100	98	N.D.	N.D.	N.D.	N.D.	N.D.
Hollyfield 2018) [11]			134	77	(57.5)	8	38	17	33	4	33	67	100	N.D.	N.D.	N.D.	N.D.	N.D.
Jaiswal 2018) [12]			192	62	(32.2)	3	33	7	100	7	100	100	92	86.9	63.2	97.6	92.3	85.4
Karuna 2019) [13]			105	76	(72.4)	2	0	0	50	2	33	100	93	94.6	85.0	98.1	94.4	94.6
Layfield 2018) [14]			164	164			11	5	19	5	40	60	93	95.2	N.D.	N.D.	N.D.	N.D.
Maleki 2019) [15]			734	333	(45.4)	5	11	8	28	3	42	82	94	N.D.	N.D.	N.D.	N.D.	N.D.
Montezuma 2018) [16]			388	104	(26.8)	7	25	33	9	2	40	50	100	95.9	62.5	100.0	100.0	95.6
Park 2018) [17]			413	413		5	20	7	0	2	26	83	100	N.D.	N.D.	N.D.	N.D.	N.D.
Pujani 2019) [18]			150	64	(42.7)	3	0	10	50	3	50	100	100	96.9	81.8	100.0	100.0	96.4
Rohilla 2017) [19]			631	94	(14.9)	3	0	17	100	7	50	N.D.	96	91.4	79.4	98.3	96.4	89.2
Sadullahoğlu 2019) [20]			459	129	(28.1)	4	22	22	60	0	14	79	90	85.8	95.1	81.0	72.2	96.9
Savant 2018) [21]			331	199	(60.1)	6	0	0	33	1	41	100	100	N.D.	N.D.	N.D.	N.D.	N.D.
Song 2019) [22]			893	429	(48.0)	8	18	14	31	2	47	79	99	N.D.	N.D.	N.D.	N.D.	N.D.
Thiryayi 2018) [23]			287	138	(48.0)	3	9	2	0	2	27	100	100	99.0	94.6	100.0	100.0	98.8
Vallonthaiel 2018) [24]			893	190	(21.3)	5	44	8	0	8	44	81	100	N.D.	84.0	96.0	92.0	89.0
Viswanathan 2018) [25]			627	373	(59.5)	6	7	7	39	5	34	93	92	N.D.	N.D.	N.D.	N.D.	N.D.
Wei 2017 (29 studies) [26]			4514	4514			25	10	N.D.	3	38	59	92	N.D.	87.0	85.0	92.0	77.0
Overall			29307	24885	(84.9)	6	16	11	36	3	40	82	96	93.2	82.9	95.1	92.0	92.1
Present Study	Patient Based	All	138	70	(50.7)	1	0	100	14	0	27	100	100	90.9	61.5	100.0	100.0	89.4
		Parotid	114	57	(50.0)	1	0	N.D.	14	0	22	100	100	93.5	62.5	100.0	100.0	92.7
		Submandibular	19	11	(57.9)	1	0	100	N.D.	0	100	100	100	71.4	50.0	100.0	100.0	60.0
		other	5	2	(40.0)	1	N.D.	N.D.	N.D.	N.D.	0	N.D.	100	N.D.	N.D.	N.D.	N.D.	N.D.
	Sample Based	All	183	90	(49.2)	1	6	100	11	0	25	100	100	91.5	64.3	100.0	100.0	90.0
		Parotid	153	69	(45.1)	1	4	N.D.	11	0	20	100	100	94.0	66.7	100.0	100.0	93.2
		Submandibular	25	15	(60.0)	1	14	100	N.D.	0	100	100	100	71.4	50.0	100.0	100.0	60.0
		other	5	2	(40.0)	1	N.D.	N.D.	N.D.	N.D.	0	N.D.	100	N.D.	N.D.	N.D.	N.D.	N.D.

Abbreviations: ND, Non-Diagnostic; NN, Non-Neoplastic; AUS, Atypia of Undetermined Significance; BM, Benign Neoplasm; SUMP, Neoplasm of Uncertain Malignant Potential; SM, Suspicious for Malignancy; MN, Malignant Neoplasm; PPV, positive predictive value; NPV, negative predictive value; N.D., not determined.
